# International travel and the risk of hospitalization with non-typhoidal *Salmonella *bacteremia. A Danish population-based cohort study, 1999-2008

**DOI:** 10.1186/1471-2334-11-277

**Published:** 2011-10-19

**Authors:** Kristoffer Koch, Brian Kristensen, Hanne M Holt, Steen Ethelberg, Kåre Mølbak, Henrik C Schønheyder

**Affiliations:** 1 Department of Clinical Microbiology, Aalborg Hospital, Aarhus University Hospital, Aalborg, Denmark; 2 Department of Clinical Microbiology, Skejby Hospital, Aarhus University Hospital, Aarhus, Denmark; 3 Department of Clinical Microbiology, Odense University Hospital, Odense, Denmark; 4 Department of Epidemiology, Statens Serum Institut, Copenhagen, Denmark

## Abstract

**Background:**

Information is sparse regarding the association between international travel and hospitalization with non-typhoidal *Salmonella *bacteremia. The aim of this study was to determine the proportion, risk factors and outcomes of travel-related non-typhoidal *Salmonella *bacteremia.

**Methods:**

We conducted a 10-year population-based cohort study of all patients hospitalized with non-typhoidal *Salmonella *bacteremia in three Danish counties (population 1.6 million). We used denominator data on Danish travellers to assess the risk per 100,000 travellers according to age and travel destination. We used patients contemporaneously diagnosed with travel-related *Salmonella *gastroenteritis as reference patients to estimate the relative risk of presenting with travel-related bacteremia as compared with gastroenteritis. To evaluate clinical outcomes, we compared patients with travel-related bacteremia and patients with domestically acquired bacteremia in terms of length of hospital stay, number of extraintestinal focal infections and mortality after 30 and 90 days.

**Results:**

We identified 311 patients hospitalized with non-typhoidal *Salmonella *bacteremia of whom 76 (24.4%) had a history of international travel. The risk of travel-related bacteremia per traveller was highest in the age groups 15-24 years (0.8/100,000 travellers) and 65 years and above (1.2/100,000 travellers). The sex- and age-adjusted relative risk of presenting with bacteremia was associated with travel to Sub-Saharan Africa (odds ratio 18.4; 95% confidence interval [6.9-49.5]), the Middle East (10.6; [2.1-53.2]) and South East Asia (4.0; [2.2-7.5]). We found high-risk countries in the same three regions when estimating the risk per traveller according to travel destination. Patients hospitalized with travel-related bacteremia had better clinical outcomes than patients with domestically acquired bacteremia, they had a shorter length of hospital stay (8 vs. 11 days), less extraintestinal focal infections (5 vs. 31 patients) and a lower risk of death within both 30 days (relative risk 0.2; [0.1-0.7]) and 90 days (0.3; [0.1-0.7]). A healthy traveller effect was a plausible explanation for the observed differences in outcomes.

**Conclusions:**

International travel is a notable risk factor for being hospitalized with non-typhoidal *Salmonella *bacteremia and the risk differs between age groups and travel destinations. Healthy travellers hospitalized with bacteremia are less likely to have poor outcomes than patients with domestically acquired bacteremia.

## Background

International travel has increased manifold during the last decades as a consequence of increasing prosperity and reduced costs. Overseas destinations have become popular holiday resorts for Europeans, e.g., Thailand was visited by 758,000 Scandinavians in 2007 [1]. As a consequence there is increasing attention to the exposure to parasitic and viral infections including malaria, hepatitis, dengue fever, and HIV-infection [2-5]. Among travel-related bacterial infections, typhoid and paratyphoid fevers have been addressed in a number of studies [6-8]. Less information is available concerning invasive infections with non-typhoidal *Salmonella enterica *serovars (henceforth *Salmonella*) although they are common causes of gastrointestinal infections in travellers. In a recent Swedish study the highest risk of gastrointestinal salmonellosis was associated with travelling to East Africa and the Indian subcontinent [9]. In a Danish cohort study from the 1980's, 18% of *Salmonella *bacteremias were associated with travel [10].

European studies on travel-related risk of *Salmonella *bacteremia are few and either clinic-based [11,12] or confined to a particular serovar or travel destination [13]. Therefore, the proportion of patients hospitalized with travel-related bacteremia remains uncertain and knowledge of potential risk factors and outcomes are lacking. Such data would be of value for providers of guidance to travellers and for clinical decision making. The objective of this study was to determine the proportion of hospitalized patients with non-typhoidal *Salmonella *bacteremia related to international travel, to assess whether the risk of bacteremia was associated with the travel destination, age or sex, and to evaluate the clinical outcome.

## Methods

### Study population

We conducted a population-based cohort study in the three Danish counties of North Jutland, Aarhus and Funen from January 1999 through December 2008 (average population 1.6 million, i.e. approximately one third of the Danish population). The demographic composition was representative for all of Denmark.

The National Health Service provides tax-supported health care free of charge to all Danish residents, provided by general practitioners and public hospitals. People with Danish citizenship or permanent residency in Denmark are uniquely identifiable by a 10-digit civil registration number, which is recorded at all contacts with the health service allowing accurate linkage between health registries.

### Data Sources

#### Local microbiological databases

In each county a single diagnostic laboratory processed all bacteriological samples submitted by general practitioners and hospitals. Information on blood isolates of *Salmonella *serovars were retrieved from the electronic laboratory information systems in each laboratory.

#### The National Registry of Enteric Pathogens (REP)

Diagnostic laboratories mandatorily report *Salmonella *infections to the national reference center at Statens Serum Institut (SSI) and this information is recorded in the REP. If a *Salmonella *serovar is isolated more than once from the same person within six months, only the first positive sample is registered in the REP. Sample type (feces, blood or another usually sterile site) is not available for all isolates. For the years 2004-2008 the database holds information on international travel provided by the local laboratory on a voluntarily basis. In 2008 full travel information in REP was obtained by telephone interviews [14]. The travel information collected by telephone interviews and notified voluntarily has been found comparable with regard to the distribution of travel destinations. We identified patients with *Salmonella *infection who were residents in any of the three counties in the REP.

#### Hospital charts

For patients hospitalized with non-typhoidal *Salmonella *bacteremia, we extracted the following information: travel history, comorbidity, any extraintestinal site of infection, dates of admission and discharge, length of stay and time of death. Comorbidity was classified according to the Charlson comorbidity index and three levels of comorbidity were defined: low (0), corresponding to patients with no recorded underlying diseases; medium (1-2) and high (≥3) [15].

#### Demographic data

Number of citizens in the three counties according to age groups were available from Statistics Denmark and used as denominators to calculate incidence rates [16].

#### Data on international travel

Statistics Denmark obtains information on international travel from a random sample of approximately 500 Danish citizens contacted by telephone each month. Information in the database includes age groups, travel destinations, month of travel, type of travel (business or pleasure), and length of travel. We restricted our analyses to pleasure travellers as business travellers may have a different travel form and risk profile. To account for a large number of short trips to neighboring countries, we included only travellers staying abroad for at least three nights. We used the information in the database to estimate travel patterns in the study population. Estimated numbers by age group and country of travel were used as denominators to calculate the risk of bacteremia per 100,000 travellers.

### Laboratory procedures

Blood culture systems and nominal volumes of blood per culture differed between regions (North Jutland County: BacT/Alert (bioMérieux, Marcy I'Etoile, France) with a blood volume of 3 × 10 mL; Aarhus County: BacT/Alert (bioMérieux, Marcy I'Etoile, France); 2 × 20 mL; Funen County: ESP (Trek Diagnostic Systems, Cleveland, Ohio, USA) until 2001, and BACTEC (Becton Dickinson, Franklin Lakes, NJ, USA) thereafter; 2 × 20 mL).

In North Jutland County fecal cultures were performed by the regional diagnostic laboratory throughout the study period [17]. In the counties of Aarhus and Funen the regional laboratories carried out the cultures from 2004 and 2006, respectively. Prior to that, fecal cultures were performed by SSI, Copenhagen.

All *Salmonella *isolates obtained locally were referred to the national reference laboratory at SSI for serotyping according to the Kauffman-White scheme [18].

### Definitions

We defined travel-related *Salmonella *bacteremia (henceforth TRB) on the basis of information in the hospital chart regarding foreign travel prior to admission and lack of any indication of domestic exposure. The remaining bacteremias were defined as domestically acquired (henceforth DAB). If a patient had visited more than one destination, we defined the main travel destination as the country where the patient had had an episode of diarrhea. If this information was missing, the last visited country was defined as the travel destination.

A patient with *Salmonella *gastroenteritis was defined as a patient recorded with non-typhoidal *Salmonella *in the REP and no blood culture isolate recorded in the local microbiological database.

### Data analysis

Clinical data retrieved from patient charts were tabulated in a spreadsheet. Relative prevalence proportions (RPP) with corresponding 95% confidence intervals were calculated to estimate differences in age, sex and comorbidity among patients with TRB compared to DAB. Risk estimates per 100,000 travellers were calculated according to travel destination and age groups (children 0-14, young adults 15-24, adults 25-44, middle-aged 45-64, and elderly ≥65 years).

In the subset of patients diagnosed in 2004-2008, we estimated the risk of TRB compared to travel-related gastroenteritis by calculation of odds ratios (ORs) with 95% confidence intervals (CI). Risk associated with travel region was estimated by logistic regression and age group (<65, ≥65 years) and gender were included as potential confounders.

STATA version 9.2 (College Station, Texas) was used for the statistical analyses.

### Ethics

The study was approved by The Danish National Board of Health and the Danish Data Protection Agency (Record no. 2008-54-0474).

## Results

### Demographic, clinical and bacteriological characteristics

During the 10-year study period a total of 350 patients were hospitalized and diagnosed with *Salmonella *bacteremia. The following cases were excluded: *Salmonella *Typhi (15), *Salmonella *Paratyphi (14) and non-residents (10), leaving 311 patients with non-typhoidal *Salmonella *bacteremia.

We identified 76 (24.4%) patients hospitalized with TRB. Most (68/76, 89%) were admitted to hospital within two weeks after returning to Denmark; patients admitted later (8/76, 11%) had had diarrhea during travel and fever after the return. Four of these patients had been treated with antibiotics by their general practitioner before hospital admission.

Table 1 presents key characteristics for patients with TRB and DAB. In the group of patients with TRB there was a majority of males and patients with TRB were younger than patients with DAB (RPP for males in TRB compared to DAB was 1.3; 95% CI[1.0-1.6]; RPP for age <65 years was 1.6; [1.4-1.9]). Seventy one percent of patients with TRB had no comorbidities according to the Charlson index compared with 29% of patients with DAB (RPP 2.4; [1.9-3.1]).

**Table 1:** Characteristics of patients with travel-related (TRB) and domestically acquired (DAB) bacteremia

Characteristics	TRB (n = 76)		DAB (n = 235)	
Age				
Median (years, range)	36	(0-79)	64	(0-96)
<65 years (numbers, percent)	64	(84.2)	123	(52.3)
≥65 years	12	(15.8)	112	(47.7)
				
Sex				
Males	49	(64.5)	118	(50.2)
				
Admitted to an infectious diseases unit^a^	29/64	(45.3)	8/163	(4.9)
				
Comorbidity index^b^				
0	54	(71.1)	69	(29.4)
1-2	11	(14.5)	91	(38.7)
≥3	11	(14.5)	75	(31.9)
				
Individuals with malignancy				
Hematological malignancies^c^	3	(3.9)	23	(9.8)
Other malignancies	6	(7.9)	36	(15.3)
				
Other comorbidities				
Dialysis^d^	0	(0.0)	3	(1.3)
Organ transplantation	0	(0.0)	2	(0.9)
Alcoholism^e^	3	(3.9)	21	(8.9)
Previous gastric surgery^f^	3	(3.9)	5	(2.1)
				
Drug therapy				
Chemotherapy & immunomodulatory agents	5	(6.6)	33	(14.0)
Corticosteroid therapy	2	(2.6)	30	(12.8)
PPI & H2-antagonist	7	(9.2)	66	(28.1)
Antacids	0	(0.0)	8	(3.4)
				
Length of hospital stay (median, IQR)	8 days	(5-11)	11 days	(6-17)
				
An extraintestinal site of infection	5	(6.6)	31	(13.2)
				
Mortality by age group				
<65 years				
30-day	3	(4.7)	11	(8.9)
90-day	4	(6.3)	12	(9.8)
≥65 years				
30-day	0	(0.0)	30	(26.8)
90-day	1	(8.3)	40	(35.7)
Overall				
30-day	3	(3.9)	41	(17.4)
90-day	5	(6.6)	52	(22.1)

^a ^Only numbers from North Jutland and Aarhus Counties.

^b ^Charlson comorbidity index.

^c ^Lymphoma, leukaemia and myeloma; Patients with myeloproliferative disease or myelodysplastic syndrome also included.

^d ^Dialysis due to complications to the bacteremia not included.

^e ^Both history of present and previous alcoholism included.

^f ^Gastrectomy, vagotomy, gastric bypass.

There were also notable differences in outcome between the two groups of patients. Patients with TRB had shorter length of hospital stay and fewer extraintestinal sites of infection than patients with DAB, e.g. osteomyelitis, infected aortic aneurysm, intraabdominal abscess, or pleural empyema. The relative risk (RR) of death within 30 days was lower among patients with TRB (RR 0.2; [0.1-0.7]; RR of death after 90 days: 0.3; [0.1-0.7]). This was due to a high mortality among patients with DAB aged 65 years and above.

*S*. Enteritidis and *S*. Thyphimurium were among the most common serovars identified from both patients with DAB and TRB, together they counted for 55% and 37%, respectively. *S*. Dublin was the second most common serovar from patients with DAB (20%), but was rare among patients with TRB. Conversely, few patients with DAB had *S*. Virchow, which accounted for 10% of the serovars from patients with TRB. In total, 34 different serovars were identified among the domestically acquired infections, and 30 among the group of travel-related infections (Table 2).

**Table 2:** Serovars isolated from patients with travel-related (TRB) and domestically acquired (DAB) bacteremia

Serovar	TRB Number (%) (n = 78)*		Serovar	DAB Number (%) (n = 235)	
					
*S*. Enteritidis	23	(29.5)	*S*. Enteritidis	84	(35.7)
*S*. Virchow	8	(10.3)	*S*. Dublin	48	(20.4)
*S*. Typhimurium	6	(7.7)	*S*. Typhimurium	46	(19.6)
*S*. Newport	4	(5.1)	*S*. Bovismorbificans	6	(2.6)
*S*. Panama	4	(5.1)	*S*. Newport	5	(2.1)
*S. *Aviana	3	(3.8)	*S. *Agona	4	(1.7)
*S. *Saint-Paul	3	(3.8)	*S. *Java	4	(1.7)
*S. *Corvallis	2	(2.6)	*S. *Oranienburg	4	(1.7)
*S. *Haifa	2	(2.6)	*S. *Infantis	3	(1.3)
*S. *Heidelberg	2	(2.6)	*S. *Chester	2	(0.9)
*S. *Java	2	(2.6)	*S. *Hadar	2	(0.9)
*S. *Poona	2	(2.6)	*S. *Heidelberg	2	(0.9)
Others	17	(21.8)	*S. *Poona	2	(0.9)
			*S. *Saint-Paul	2	(0.9)
			*S. *Thompson	2	(0.9)
			Others	19	(8.1)

*Two patients had double infections with different serovars.

### Incidence and trends

Overall, the proportion of TRB was higher in the second 5-year period, 28.3% [range 25.7-32.5%], compared with the first 5-year period, 20.8% [range 15.6-28.2%] (RPP 1993-2003 vs. 2004-2008: 1.4; [0.9-2.0]). Higher annual numbers of DAB during the first period contributed to this difference. The overall incidence rate of TRB was 0.5/100,000 person-years, with the highest incidence rate in the age group 15-24 years (0.8/100,000 person-years).

### Seasonal patterns

**Figure 1:**
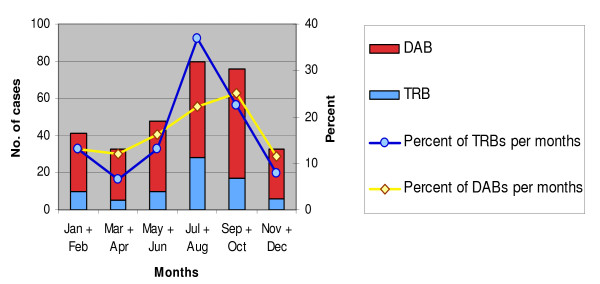
**Seasonal pattern of travel-related (TRB) and domestically acquired (DAB) bacteremias, 1999-2008**.

Both DABs and TRBs showed a seasonal variation when estimated within six periods of two months each. Most cases of DABs were found in the autumn months, September-October (59/235, 25%) and summer months, July-August (52/235, 22%). Travel-associated cases had a distinct peak in July-August (28/76, 37%), which were the months with most travellers (Figure 1).

### Travel duration and destinations

Information on the duration of stay was available in (53/76, 70%) of the TRB cases. The majority of patients had travelled for less than three weeks (40/53, 75%), giving a median stay of fourteen days (interquartile range (IQR) 9-21 days).

Patients hospitalized with TRB had travelled to 30 different countries. The highest numbers of TRBs were contracted in Thailand (16), followed by Greece (6), Spain (6), Egypt (5) and Turkey (5). Using data on travel patterns for Danish tourists as denominator, we found that the risk of hospitalization with *Salmonella *bacteremia were different between age groups and travel destinations (Table 3).

**Table 3:** Estimated number of travellers and risk of bacteremia per 100,000 travellers by age groups and country (> 3 night stay)

Age group/Country*	Number of travel-related bacteremias (TRB) (1999-2008)	Estimated number of travellers† (1999-2008)	Risk per 100,000 travellers (95% CI)	
				
0-14 years	13	3,300,000	0.4	(0.2-0.7)
15-24 years	15	1,800,000	0.8	(0.5-1.4)
25-44 years	16	3,730,000	0.4	(0.3-0.7)
45-64 years	20	3,860,000	0.5	(0.3-0.8)
≥65 years	12	980,000	1.2	(0.7-2.2)
				
Spain	6	1,340,000	0.4	(0.2-1.0)
Sweden	1	780,000	0.1	(0.0-0.9)
Germany	3	690,000	0.4	(0.1-1.4)
Greece	6	670,000	0.9	(0.4-2.0)
Turkey	5	440,000	1.1	(0.5-2.8)
Poland	2	340,000	0.6	(0.1-2.4)
Portugal	3	310,000	1.0	(0.3-3.0)
Thailand	16	300,000	5.3	(3.3-8.7)
Egypt	5	260,000	1.9	(0.8-4.7)
Czech Republic	2	220,000	0.9	(0.2-3.7)
Bulgaria	2	130,000	1.5	(0.4-6.3)
Croatia	1	90,000	1.1	(0.2-8.1)
Vietnam	4	75,000	5.3	(1.9-15.0)
India	1	75,000	1.3	(0.2-9.7)
Mexico	1	60,000	1.7	(0.2-12.3)
United Arab Emirates	1	35,000	2.9	(0.4-21.7)
Ukraine	1	35,000	2.9	(0.4-21.5)
China	1	30,000	3.3	(0.4-25.5)
Tanzania	1	25,000	4.0	(0.5-31.2)
Malta	1	8000	12.5	(1.4-114.6)
Malaysia	1	7000	14.3	(1.5-137.3)
Ghana	2	6000	33.3	(5.6-199.5)
Senegal	1	6000	16.7	(1.7-160.2)

*Number of travellers to Kenya, Cambodia, Guatemala, Indonesia, Iraq, Somalia and Uganda were not available. Three cases of TRB were contracted in Kenya and one case in each of the other countries.

† Numbers for the age group 0-14 years were based on the proportion of adults travelling with children

### Relative risk of TRB compared to travel-related gastroenteritis 2004-2008

From 2004-2008, we identified 2865 patients with *Salmonella *gastroenteritis in the REP after exclusion of patients with bacteremia and cases of typhoid or paratyphoid fever. All patients were residents in one of the three counties at the time of positive fecal cultures. Of these, 527 (18.4%) patients were recorded as having travel-related *Salmonella *gastroenteritis.

We found Sub-Saharan Africa, the Middle East, and South East Asia to be high-risk regions for contracting TRB when compared to contemporary cases of travel-associated *Salmonella *gastroenteritis. An increased risk was also found in the age group of 65 years and above, but there were no difference related to sex (Table 4).

**Table 4:** Relative risk of travel-related bacteremia (TRB) compared to travel-related gastroenteritis, 2004-2008*

Variable	Number of TRBs (n = 43)	Odds ratio†	95% CI
Male	28	1.0	0.7-1.4
Age ≥65 years	6	5.3	3.7-7.6
Travel region‡	7	18.4	6.9-49.5
Sub-Saharan Africa	2	10.6	2.1-53.2
Middle East	14	4.0	2.2-7.5
South East Asia			

*Both voluntary and interview-based information were available during this period

†Adjusted for other risk factors in the model

‡Travel regions included in the analyses: East Asia & India, Eastern Europe & Russia, Middle East, Northern Africa, South East Asia, Southern Europe & Eastern Mediterranean, Sub-Saharan Africa, The Americas, Western Europe & Nordic countries

## Discussion

In this cohort study, we found that the overall incidence of patients hospitalized with travel-related non-typhoidal *Salmonella *bacteremia was low (0.5/100,000 person-years). Still, one in four cases hospitalized with *Salmonella *bacteremia in Denmark was associated with travel. The risk was most pronounced for the elderly and the young adult travellers and for travellers to countries in the regions of Sub-Saharan Africa, the Middle East and South East Asia. However, patients with travel-related infections were less likely to have poor outcomes than patients with domestically acquired infections. Our data also suggested an increasing trend in the proportion of TRBs, which is further supported by the previous Danish population-based study from the 1980s [10]. One crucial factor is probably the major reduction in the incidence of domestically acquired *Salmonella *infections which followed implementation of a national *Salmonella *control program in 1997 [19,20]. During the last three decades the popularity of overseas travel has been increasing and together these two trends are likely to have changed the national 'balance sheet' for invasive *Salmonella *infections.

Our study has a number of important limitations. First, our study design only allowed for detection of *Salmonella *infections among hospitalized patients and our results were therefore based on estimates of the risk of being hospitalized with bacteremia and not on the risk of falling ill for which no data were available. Although, we believe that our study included all severe cases of invasive *Salmonella *infections, patients with a fever and a history of international travel may have been hospitalized at a lower threshold of clinical suspicion than patients with domestically acquired infections. This would lead to a potential overestimation of the proportion of TRB as compared with DABs. Second, we only included patients with bacteremia diagnosed after their return to Denmark. Travellers who were diagnosed and treated for bacteremia abroad therefore did not appear in the data. Admission and treatment at local hospitals may be more likely for travellers staying abroad for extended periods and the risk of bacteremia may therefore be biased towards destinations typically associated with shorter stays. Third, we were not able to validate the travel information given in hospital charts which relied on the clinicians' notes. If bias were introduced, it would most likely be in the form of 'missing data' on foreign travel. This would entail a potential underestimation of the proportion of TRBs. However, when considering the potential for misclassification of the travel information, we still believe that the different risk estimates reflect genuine differences in exposure.

Previous studies have shown the greatest risk of non-typhoidal *Salmonella *bacteremia at the extremes of age and we expected to find the same age distribution among patients with TRB [21]. Several predisposing comorbidities are acknowledged risk factors for *Salmonella *bacteremia, e.g. chronic or immunosuppressing conditions, including malignancy, rheumatological disease, liver disease, and diabetes [22-24], and it is likely that the elevated risk of TRB in the elderly reflected an increased prevalence of comorbidities in this age group. We used the Charlson comorbidity index as a summary measure of the patients' comorbidities, which includes 19 major disease categories, several of which are known risk factors for *Salmonella *bacteremia. Comparison of the burden of comorbidity in patients ≥65 years with that of patients <65 years (data not included in the results) showed that 67% (8/12) of patients ≥65 years had one or more diseases registered in the Charlson index compared with only 22% (14/64) in patients <65 years (RPP for ≥1 comorbid disease: 3.0 [95% CI: 1.7-5.6]).

A previous study suggested that accommodation, travel characteristics, and dietary hygiene are important factors for the evaluation of traveller's risk of diarrhea [25] and that the higher risk of traveller's diarrhea found in young adults may be associated with ingestion of larger volumes of potentially contaminated food and an adventurous lifestyle in this age group [26]. These behavioural factors would be important for the higher risk of *Salmonella *bacteremia in young adults, although the risk was only marginally higher than that reported for other age groups. A formal analysis was not possible as we had no data on behavioral factors or precise data on travel form.

Comparison of the characteristics between patients hospitalized with TRB and DAB (Table 1) illustrated that the two groups were fundamentally different. There were major differences in clinical outcomes between the groups. Thus, patients with TRB had a shorter length of hospital stay, fewer complications, and lower mortality than patients with DAB. The explanation for the latter observation is likely to be a "healthy traveller effect" as illustrated by the lower prevalence of comorbidities in the group of patients infected abroad. This is consistent with a Swedish study on *Salmonella*-associated deaths where persons infected domestically had a much more severe prognosis than those infected abroad [27].

Earlier studies have shown that the distribution of serovars may vary much by geographic location and time [28,29]. Overall, *S*. Enteritidis was the predominant serovar in Denmark throughout the study period, even though *S*. Typhimurium was the most frequent serovar in the last year of the study (2008) because of an unprecedented, large nationwide outbreak [14]. *S*. Enteritidis was also the most common serovar in Europe and dominant in most of the travel regions [9]. This is in accordance with our finding that *S*. Enteritidis was the most frequent serovar both in patients with TRB and DAB. *S*. Virchow and *S*. Dublin were the second most common serovars isolated from patients with TRB or DAB, even though *S*. Typhimurium were a much more common serovar in most regions of the world. Both *S*. Virchow and *S*. Dublin show a higher propensity for causing bacteremia and more severe infections than *S*. Typhimurium [21,30]. This suggests that the distribution of serovars was due to both the overall abundance of serovars as well as the exposure to more invasive serovars.

## Conclusions

International travel is an acknowledged risk factor for non-typhoidal *Salmonella *gastroenteritis, however, little is known regarding the risk of potentially more severe invasive *Salmonella *infections. This study demonstrated that international travel is a notable risk factor for being hospitalized with non-typhoidal *Salmonella *bacteremia and the risk differs among travellers according to age and travel destination. Still, the overall risk is low and healthy travellers with bacteremia are less likely to have poor outcomes compared to patients with domestically acquired bacteremia. Our findings may be useful for the clinical evaluation of travellers presenting with fever and may also support pre-travel counselling.

## Competing interests

The authors declare that they have no competing interests.

## Authors' contributions

KK participated in the study design, was responsible for data collection, statistical analysis and interpretation of data and drafted the manuscript. BK and HMH assisted in data collection and interpretation of data. SE and KM participated in the design, data collection and analysis and critically reviewed the manuscript. HCS was responsible for the study design and assisted with data collection, statistical analysis, interpretation of data and drafting the manuscript. All authors reviewed and approved the final manuscript.

## Pre-publication history

The pre-publication history for this paper can be accessed here:

http://www.biomedcentral.com/1471-2334/11/277/prepub

## References

[B1] Tourism Authority of Thailand (TAT), Tourism Statistics http://www2.tat.or.th/stat/web/static_index.php

[B2] Askling HH Rombo L Andersson Y Martin S Ekdahl K Hepatitis A risk in travelers J Travel Med 2009 16 233 238 10.1111/j.1708-8305.2009.00307.x 19674261

[B3] Leggat PA Zwar NA Hudson BJ Hepatitis B risks and immunisation coverage amongst Australians travelling to southeast Asia and east Asia Travel Med Infect Dis 2009 7 344 349 10.1016/j.tmaid.2009.03.008 19945011

[B4] Cobelens FG Groen J Osterhaus AD Leentvaar-Kuipers A Wertheim-van Dillen PM Kager PA Incidence and risk factors of probable dengue virus infection among Dutch travellers to Asia Trop Med Int Health 2002 7 331 338 10.1046/j.1365-3156.2002.00864.x 11952949

[B5] Thomson MM Najera R Travel and the introduction of human immunodeficiency virus type 1 non-B subtype genetic forms into Western countries Clin Infect Dis 2001 32 1732 1737 10.1086/320764 11360216

[B6] Lynch MF Blanton EM Bulens S Polyak C Vojdani J Stevenson J Medalla F Barzilay E Joyce K Barrett T Mintz ED Typhoid fever in the United States, 1999-2006 JAMA 2009 302 859 865 10.1001/jama.2009.1229 19706859

[B7] Lester A Mygind O Jensen KT Jarlov JO Schønheyder HC [Typhoid and paratyphoid fever in Denmark 1986-1990. Epidemiologic aspects and the extent of bacteriological follow-up of patients] Ugeskr Laeger 1994 156 3770 3775 8059456

[B8] Ekdahl K de JB Andersson Y Risk of travel-associated typhoid and paratyphoid fevers in various regions J Travel Med 2005 12 197 204 16086894 10.2310/7060.2005.12405

[B9] Ekdahl K de JB Wollin R Andersson Y Travel-associated non-typhoidal salmonellosis: geographical and seasonal differences and serotype distribution Clin Microbiol Infect 2005 11 138 144 10.1111/j.1469-0691.2004.01045.x 15679488

[B10] Lester A Eriksen NH Nielsen H Nielsen PB Friis-Møller A Bruun B Scheibel J Gaarslev K Kolmos HJ Non-typhoid Salmonella bacteraemia in Greater Copenhagen 1984 to 1988 Eur J Clin Microbiol Infect Dis 1991 10 486 490 10.1007/BF01963934 1915383

[B11] Ispahani P Slack RC Enteric fever and other extraintestinal salmonellosis in University Hospital, Nottingham, UK, between 1980 and 1997 Eur J Clin Microbiol Infect Dis 2000 19 679 687 10.1007/s100960000341 11057501

[B12] Matheson N Kingsley RA Sturgess K Aliyu SH Wain J Dougan G Cooke FJ Ten years experience of Salmonella infections in Cambridge, UK J Infect 2010 60 21 25 10.1016/j.jinf.2009.09.016 19819256

[B13] Sirichote P Hasman H Pulsrikarn C Schønheyder HC Samulioniene J Pornruangmong S Bangtrakulnonth A Aarestrup FM Hendriksen RS Molecular characterization of extended-spectrum cephalosporinase-producing Salmonella enterica serovar Choleraesuis isolates from patients in Thailand and Denmark J Clin Microbiol 2010 48 883 888 10.1128/JCM.01792-09 20032253 PMC2832443

[B14] Ethelberg S Müller L Mølbak K Nielsen EM [Salmonella and campylobacter infections in 2008] Ugeskr Laeger 2010 172 1451 1455 20470656

[B15] Charlson ME Pompei P Ales KL MacKenzie CR A new method of classifying prognostic comorbidity in longitudinal studies: development and validation J Chronic Dis 1987 40 373 383 10.1016/0021-9681(87)90171-8 3558716

[B16] Statistics Denmark, StatBank Denmark http://www.statistikbanken.dk

[B17] Blom M Meyer A Gerner-Smidt P Gaarslev K Espersen F Evaluation of Statens Serum Institut enteric medium for detection of enteric pathogens J Clin Microbiol 1999 37 2312 2316 10364603 10.1128/jcm.37.7.2312-2316.1999 PMC85145

[B18] Popoff MY Le Minor L Antigenic formulas of the Salmonella serovars 1997 WHO Collaborating Centre for Reference and Research on Salmonalle, Institut Pasteur, Paris, France

[B19] Wegener HC Hald T Lo Fo WD Madsen M Korsgaard H Bager F Gerner-Smidt P Mølbak K Salmonella control programs in Denmark Emerg Infect Dis 2003 9 774 780 12890316 10.3201/eid0907.030024 PMC3023435

[B20] Korsgaard H Madsen M Feld NC Mygind J Hald T The effects, costs and benefits of Salmonella control in the Danish table-egg sector Epidemiol Infect 2009 137 828 836 10.1017/S0950268808000903 18644168

[B21] Jones TF Ingram LA Cieslak PR Vugia DJ Tobin-D'Angelo M Hurd S Medus C Cronquist A Angulo FJ Salmonellosis outcomes differ substantially by serotype J Infect Dis 2008 198 109 114 10.1086/588823 18462137

[B22] Dhanoa A Fatt QK Non-typhoidal Salmonella bacteraemia: epidemiology, clinical characteristics and its' association with severe immunosuppression Ann Clin Microbiol Antimicrob 2009 8 15 10.1186/1476-0711-8-15 19445730 PMC2689172

[B23] Gordon MA Salmonella infections in immunocompromised adults J Infect 2008 56 413 422 10.1016/j.jinf.2008.03.012 18474400

[B24] Hsu RB Lin FY Risk factors for bacteraemia and endovascular infection due to non-typhoid salmonella: a reappraisal QJM 2005 98 821 827 10.1093/qjmed/hci126 16203825

[B25] Kollaritsch H Traveller's diarrhea among Austrian tourists in warm climate countries: I. Epidemiology Eur J Epidemiol 1989 5 74 81 10.1007/BF00145049 2785058

[B26] De Las CC Adachi J Dupont H Review article: travellers' diarrhoea Aliment Pharmacol Ther 1999 13 1373 1378 10.1046/j.1365-2036.1999.00638.x 10571591

[B27] Ternhag A Torner A Ekdahl K Giesecke J Salmonella-associated deaths, Sweden, 1997-2003 Emerg Infect Dis 2006 12 337 339 16494768 10.3201/eid1202.050867 PMC3373115

[B28] Hendriksen RS Bangtrakulnonth A Pulsrikarn C Pornruangwong S Noppornphan G Emborg HD Aarestrup FM Risk factors and epidemiology of the ten most common Salmonella serovars from patients in Thailand: 2002-2007 Foodborne Pathog Dis 2009 6 1009 1019 10.1089/fpd.2008.0245 19735204

[B29] de Jong B Ekdahl K The comparative burden of salmonellosis in the European Union member states, associated and candidate countries BMC Public Health 2006 6 4 10.1186/1471-2458-6-4 16403230 PMC1352352

[B30] Schønheyder HC Ejlertsen T Survey of extraintestinal nontyphoid Salmonella infections in a Danish region. Inverse relation of invasiveness to frequency of isolation APMIS 1995 103 686 688 10.1111/j.1699-0463.1995.tb01423.x 7488391

